# Periprosthetic pathologic fracture following tibial Echinoccocosis: A case report

**DOI:** 10.1016/j.ijscr.2018.08.045

**Published:** 2018-08-29

**Authors:** Germano Nascimento, Carmen Silva, Raquel Marques, Carlos Silva, João Francisco Oliveira, Jorge Santos, João Leiria

**Affiliations:** Hospital Dr. Manoel Constâncio, Lg. Eng. Bioucas, 2200-200, Abrantes, Portugal

**Keywords:** TKA, total knee arthroplasty, HC, hydatid cyst, Echinoccocosis, Hydatid cyst, Total knee replacement, Pathologic fracture

## Abstract

**Introduction:**

Hydatid disease in humans caused by the parasitic tapeworm *Echinococcus granulosus* has an osseous involvement of about 0.5%–2.5% of all cases in humans. The location of hydatid cysts in the tibia is seldom described in the medical literature, and its diagnosis and treatment is challenging.

**Case report:**

This paper presents a patient, with a long term, well tolerated, bilateral total knee arthroplasty (TKA), treated at our clinic, with a recent history of pain and oedema in her left upper leg. After achieving a, radiologically and histhopathologically, well documented, diagnosis of Echinoccocosis lesion in her left proximal tibia, a surgical intervention was planed, a wide resection of the cyst performed, and a specific anti-helmintic therapy was instituted. Four years later, she returns to our observation complaining of pain and unable to bear weight on her left knee, from which a pathologic fracture, adjacent to the tibial component, was diagnosed.

After surgical debridement of the newly advanced hydatid cyst growth, the TKA was revised, and due to the tibial component failure and the femoral implant loosening, a semi-constrained total knee revision arthroplasty was executed. Functional outcome was excellent.

**Conclusion:**

Although challenges in treatment of musculoskeletal hydatid cysts (HC) lesions have been documented, data regarding the musculoskeletal HC lesions resembling tumor is scarce, and those resulting in a prosthetic failure have not been published. The authors intend to add data concerning the therapeutic approach to this entity.

## Highlights

• Cystic Echinococcus in the bone is rare, comprising 0.5%–2.5% of all human hydatidosis.
• Association of Hydatid disease to pathologic fractures envolving knee prosthesis have not yet been described.
• A combined surgical and medical approach is of paramount importance to avoid recurrence.
• Resulting osseus defects is challenging and require specific prosthesis when treating this entity.

## 1 Introduction

Cystic echinococcus or hydatidosis is an anthrapozoonotic, parasitic, infection of humans and animals. This parasite has 12 different types, though 4 types are known to cause disease in humans: *Echinococcus granulosus*, *Echinococcus multilocularis*, *Echinococcus vogeli*, and *Echinococcus oligarthrus* [1,2]. Hydatid disease may develop in almost any part of the body, whilst bone localisation is rare comprising 0.5%–2.5% of all human hydatidosis [3]. The strong structure of osseous tissue limits the growth of the hydatid cyst, which spreads along medullar and trabecular channels. The disease affects the long bones, vertebral column, pelvis, and costae in order from least to most affected region. Because lesions develop very slowly, most patients with musculoskeletal hydatid disease only present in adulthood [4]. Early diagnosis is primarily based on X-ray findings which are not specific to the disease [5]. Clinical presentation is characterized by pain and edema. However, large lesions may present initially as pathologic fractures. Patients usually present at an advanced stage of the disease and, therefore, treatment is difficult and recurrence is common. [[6], [7], [8]] Although longterm survival is possible, the disease is not easy to eradicate and may be impossible to cure due to complex and difficult resection. The authors present a case that addresses a surgical complication due to this entity, which is reported in line with the SCARE criteria [9].

## 2 Case presentation

**Fig. 1:**
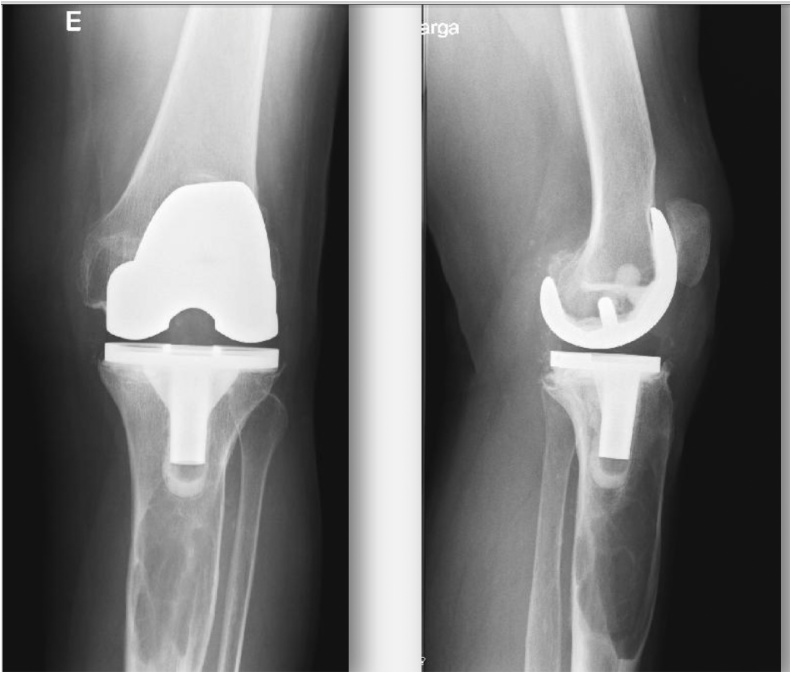
Antero-posterior and lateral x-ray view of the patient’s left knee. Please note the cystic lesion in the proximal tibial metaphysis.

In 2010 a 75 year-old female patient presented at our clinic with a 2-year history of pain and recent emergence of a discharging sinus at her left upper leg. She had a history of bilateral gonarthrosis and underwent elective right and left knee total arthroplasty 5 years before. The procedures and the post-operative follow-up were uneventful. Her physical examination revealed slight swelling and tenderness with a mild seropurulent discharge on the antero-lateral aspect on her proximal left leg. There was no other systemic complaint. Her personal and family histories were unrevealing. There was no history of fever, trauma, previous tuberculosis or bone tumors. Lower limb x-rays were performed and the radiographic examination revealed a well demarcated cystic structure in her left tibia, 4 cm below the distal tibial component of the knee arthroplasty (Fig. 1). A purulent sample was collected and sent for microbiological study, after which, to better investigate the nature of the cyst, an incisional biopsy of the lesion was performed, and the sample subjected to histopathologic examination. The laboratory study isolated *Pseudomonas aeruginosa*, and appropriate antibiotics where then administered according to the susceptibility test carried out. Pathology results revealed hydatid cyst of the tibia. Segmental resection was planned, and the surgical approach revealed a diaphyseal cyst adherent to the surrounding tissues, which were markedly oedematous, with multiple membranous whitish tissues in aggregation. Fluid was aspirated from the cyst, and the sample was sent for microbiology and serology tests. After curettage of the lesion and power-pulse lavage, povidone-iodine-alcohol solution was injected. Due to the fragility of the remaining tibial diaphysis, an external fixator was applied. Microscopy confirmed the diagnosis and revealed osseous tissue with hyaline and germinative membranes, lymphocytes, and monocytes.

Albendazole and praziquantel, antihelminthic drugs, at doses of 10 and 25 mg/kg, respectively, were started. The patient recovered uneventfully and was discharged shortly after the procedure. She was clinically and radiologically evaluated in the following months, revealing progressive bone growth, remodeling and consolidation, having the external fixator extracted 6 months after the initial procedure.

The patient then remained exempt of pain, swelling or other complications, until May 2014, when suddenly she comes to our clinic, complaining of pain and unable to bear weight on her left knee.

**Fig. 2:**
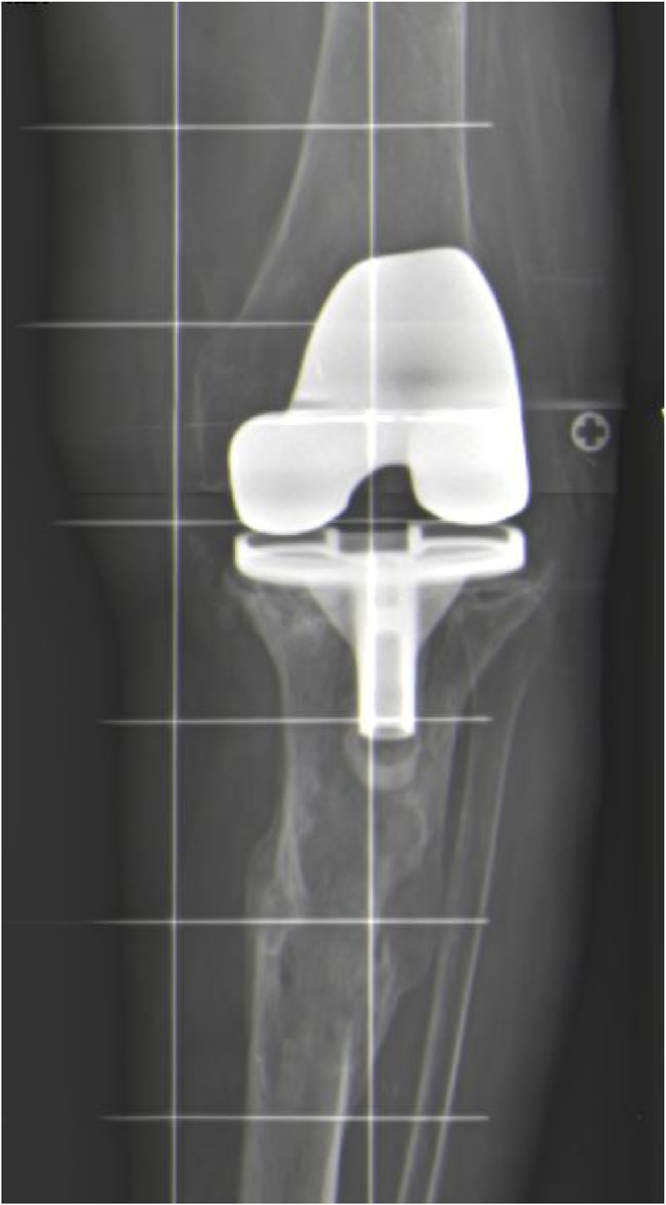
Periprosthetic pathologic fracture of the left tibia. An osteolytic pattern due to an evolved Hydatid Cyst is visible.

**Fig. 3:**
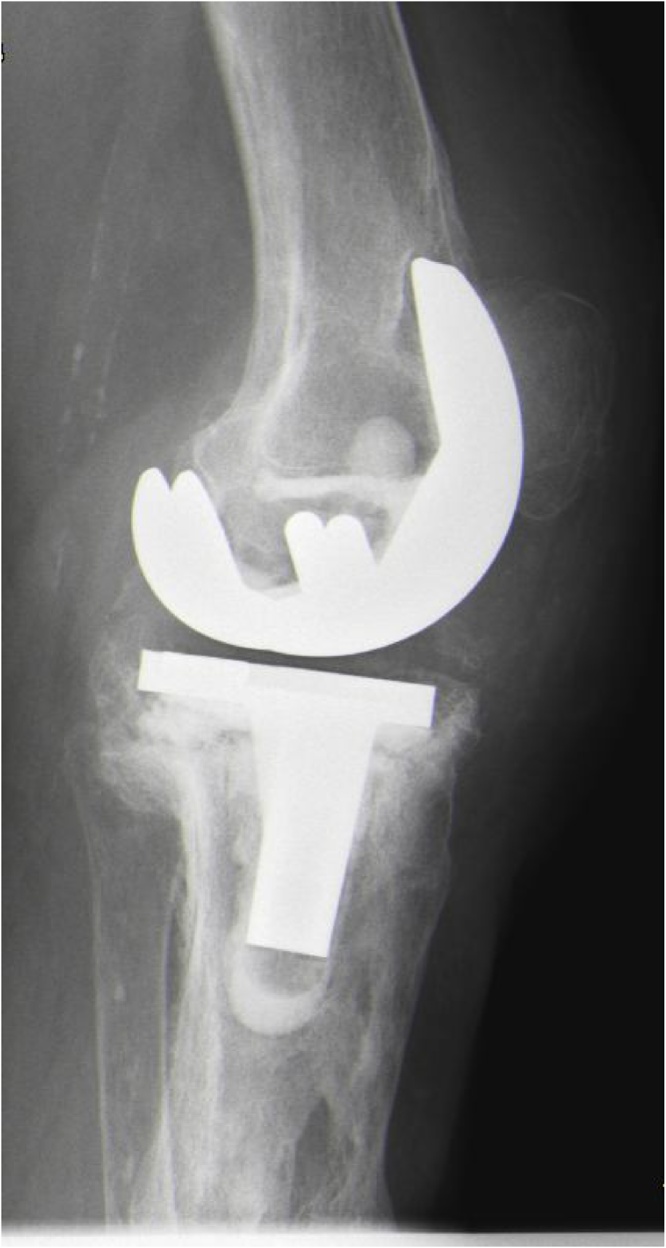
Lateral x-ray view of the total knee arthroplasty of the left knee. Extensive bone destruction is present in the proximal tibia. Loosening signs of the femoral component are present.

**Fig. 4:**
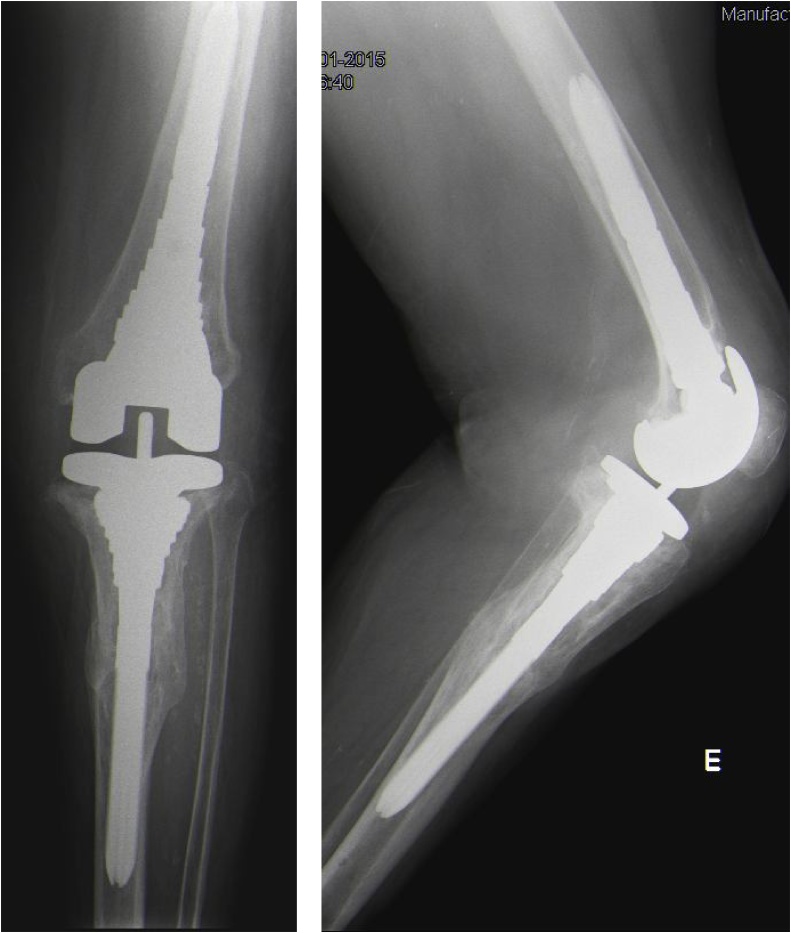
Antero-posterior and lateral x-ray view of the semi-constrained revision total knee arthroplasty with prolonged stems into the femoral and tibial medullary canal.

X-ray studies revealed a pathologic periprosthetic fracture, below the tibial component, resulting from an extension of the previously treated hydatic cyst (Fig. 2). A treatment plan was performed, and our patient underwent surgical intervention. Both of the total knee arthoplasty components exhibited signs of loosening (Fig. 3), and after extraction of them, a total revision knee arthroplasty was performed (Fig. 4). The cancellous bone loss in the tibial component was considerable, and this defect was addressed by autologous bone graft implemention around the tibial stem and plateau. The patient functional outcome was excellent. She recovered motility of her left knee, and now more than 24 months has passed and she is fully weight bearing with no pain, knee instability or discomfort.

## 3 Discussion

Periprosthetic fractures around the knee can be seen in femur, tibia and patella after total knee arthroplasty (TKA) [10]. The treatment of these fractures can be difficult since they occur in elderly patients with poor bone stock and poor healing capacity. Supracondylar femoral fractures are the most common type of periprosthetic fractures observed after TKA. Yet, periprosthetic tibial fractures after TKA are rarely seen [11].

However, there have been, to our knowledge, no reported cases of tibial component failure following a fracture due to an Hydatid Cyst of the proximal tibia.

The case presented demonstrates how Echinococcosis develops via exogenous vesiculation in bones, eventually leading to mechanical weakening and destruction of the bone trabeculae. The lesion was infiltrative in nature and progressed slowly, culminating in a pathologic fracture below the tibial component of the knee arthroplasty.

We found that the pathological fracture was associated with accelerated wear and more extensive osteolysis. Both lead to poor prosthetic survival, as seen with the loosening of the femoral component.

In the present patient, severe bone defects and lateral laxity due to huge cystic lesions compelled us to use a semi-constrained type prosthesis with a long stem. In this case, wide, radical surgical excision of the cyst required removal of most of the proximal metaphysis, and hence the implantation of this type of prosthesis, as seen in other published work [12]. The bone defect was primarily treated with autologous grafting complemented with polymethyl methacrylate cement on the tibial plateau site placement. The exothermic reaction of cementing can also contribute in preventing recurrence, as referred in some published literature [13,14].

Literature supports a combined surgical and medical approach to cases of musculoskeletal infection with Echinococcus, although the specific duration of antihelminthic therapy is yet to be determined [15,16]. We believe that wide ressection margins can significantly diminish the rate of local recurrence, especially when associated with intraoperative instilation of a povidone-iodine-alcohol solution, or furthermore with hypertonic saline as some evidence regarding this technique may suggest [17].

In this case, the treatment provided the patient with relief of pain and stable daily life. However, the follow-up period is short; thus, a longer-term follow-up is needed to investigate the real clinical usefulness of the treatment, and moreover, to evaluate the possibility of recurrence of the disease with respect to its known high recidivism rate.

## 4 Conclusion

Locally aggressive, benign bone tumors require specific techniques to avoid recurrence, thus the experience from their treatment can be used as an analogy for the treatment of musculoskeletal hydatidosis. Wide resection is essential to achieve local control and reduce the recurrence rate, and the massive defect presents a significant challenge to the reconstructive orthopedic surgeon.

Prompt diagnosis is of paramount importance for preventing destruction and complications and early treatment is essential if the affected bone is to be fully eradicated and the healthy tissue salvaged. Complete surgical excision is the treatment of choice for osseous hydatid disease.

This case report supports the use of massive bone autograft as a valid reconstruction technique after extensive bone resection as a result of hydatidic disease.

## Conflicts of interest

The authors declare that they have no conflict of interest.

## Funding

The authors declare there was no funding in collection, analysis, interpretation of data, writing and submitting this manuscript.

## Ethical approval

The ethics committee of our institution (Centro Hospitalar do Médio Tejo, E.P.E) has approved the submission and publication of this article.

## Consent

A written and signed consent was obtained from the patient and a copy is available for review by the Editor-in-Chief of this journal on request”.

## Author contribution

All the authors have been involved in the creation of this manuscript.

Dra Carmen Silva and Dr. Jorge Santos have clinically evaluated the patient, which was thereafter treated, both medically and surgically. Dr João Leiria also guided the strategy of treatment and leaded the revision procedure.

Dr. Germano Nascimento, Dra Raquel Marques, Dr. João Francisco and Dr. Carlos Silva, made all the literature research, and draft of the manuscript.

## Registration of research studies

Registered in ClinicalTrials.gov Identifier: NCT03622346.

## Guarantor

Dr Germano Nascimento.

## Provenance and peer review

Not commissioned, externally peer-reviewed.
